# Genetic polymorphism in *Methylenetetrahydrofolate Reductase chloride transport protein 6* (*MTHFR CLCN6*) gene is associated with keratinocyte skin cancer in a cohort of renal transplant recipients

**DOI:** 10.1002/ski2.95

**Published:** 2022-02-02

**Authors:** L. Griffin, L. Ho, R. J. Akhurst, S. T. Arron, J. M. E. Boggs, P. Conlon, P. O’Kelly, A. E. Toland, E. H. Epstein, A. Balmain, B. C. Bastian, F. J. Moloney, G. M. Murphy, M. E. Laing

**Affiliations:** ^1^ Department of Dermatology University Hospital Galway Galway Ireland; ^2^ Department of Dermatology Beaumont Hospital Dublin 9 Ireland; ^3^ Helen Diller Family Comprehensive Cancer Center University of California San Francisco California USA; ^4^ Department of Nephrology Beaumont Hospital Dublin 9 Ireland; ^5^ Department of Molecular Virology, Immunology and Medical Genetics Comprehensive Cancer Centre Ohio State University Columbus Ohio USA; ^6^ Department of Medicine National University of Ireland Galway Ireland

## Abstract

**Background:**

Renal transplant recipients (RTRs) are at increased risk of keratinocyte cancer (KC), especially cutaneous squamous cell carcinoma (cSCC). Previous studies identified a genetic variant of the *Methylenetetrahydrofolate Reductase* (*MTHFR*) gene, C677T, which conferred a risk for diagnosis of cSCC in Irish RTRs.

**Objective:**

We sought to find further genetic variation in *MTHFR* and overlap genes that may be associated with a diagnosis of KC in RTRs.

**Methods:**

Genotyping of a combined RTR population (*n* = 821) from two centres, Ireland (*n* = 546) and the USA (*n* = 275), was performed. This included 290 RTRs with KC and 444 without. Eleven single nucleotide polymorphisms (SNPs) in the *MTHFR* gene and seven in the overlap gene *MTHFR Chloride transport protein 6* (*CLCN6)* were evaluated and association explored by time to event analysis (from transplant to first KC) using Cox proportional hazards model.

**Results:**

Polymorphism at *MTHFR CLCN6* (rs9651118) was significantly associated with KC in RTRs (HR 1.50, 95% CI 1.17–1.91, *p* < 0.00061) and cSCC (HR 1.63, 95% CI 1.14–2.34, *p* = 0.007). A separate SNP, *MTHFR* C677T, was also significantly associated with KC in the Irish population (HR 1.31, 95% CI 1.05–1.63, *p* = 0.016), but not American RTRs.

**Conclusions:**

We report the association of a SNP in the *MTHFR* overlap gene, *CLCN6* and KC in a combined RTR population. While the exact function of *CLCN6* is not known, it is proposed to be involved in folate availability. Future applications could include incorporation in a polygenic risk score for KC in RTRs to help identify those at increased risk beyond traditional risk factor assessment.

## Graphical abstract

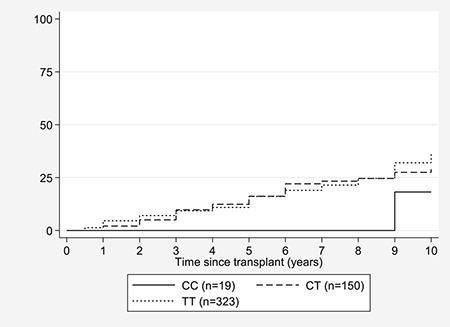

Time from date of transplant to development of keratinocyte skin cancer (Irish cohort *n* = 500) *CLCN6*. CC (usual) allele is solid line and heterozygous or homozygous variants for the *MTHFR CLCN6* (rs9651118) polymorphism are indicated here by the broken lines.

1

**What is already known about this topic?**

Changes in genomic DNA methylation are associated with cancer.^1^ Renal transplant recipients (RTRs) are at high risk from cutaneous squamous cell carcinoma (cSCC). We previously identified a genetic variant in the *MTHFR* gene (C667T rs1801133) involved in the methylation pathway which conferred a risk for the diagnosis of cSCC in Irish RTRs.^2^, ^3^ A functional was also demonstrated between this polymorphism and the methylation pathway in SCC using pyrosequencing.^3^

**What does this study add?**

We report another genetic variant in the *MTHFR* overlap gene, *CLCN6* (rs9651118) associated with cSCC in Irish and American RTRs. While the exact function of *CLCN6* is unknown, it is thought to participate in regulation of folate binding. Our findings raise the possibility of a potential SNP for inclusion in a polygenic risk score and suggests a role for folate measurement in certain patient populations.

## 2 INTRODUCTION

Renal transplant recipients (RTRs) have an increased risk of all skin cancers, especially keratinocyte cancer (i.e. cutaneous squamous cell carcinoma, cSCC, and basal cell carcinoma, BCC). They are reported to have 60–250 times the rate of cSCC as the general population and their tumours tend to have more a more aggressive course.^4^, ^5^ BCC incidence is increased to a lesser extent. Management of cSCC remains primarily surgical with additional radiotherapy and chemotherapy reserved for specific circumstances. Despite recent advances in treatment of advanced metastatic cSCC with immunotherapy in the non‐transplant population, challenges remain in solid organ recipients due to the risk of graft rejection with use of such medications. Established risk factors for the development of Keratinocyte Cancer (KC) such as male sex, advancing age, duration and type of immunosuppression, low Fitzpatrick skin type, cumulative lifetime sun exposure and more recently field cancerization have been well described.^6^ Yet, there are some RTRS who get KC earlier than others suggesting that something other than traditional risk factors contributes to overall risk. The role of genetics and epigenetics have increasing been recognized in cancer pathogenesis. Epigenetics, the study of how behaviours and environment can cause changes in gene expression, can change how the body reads a DNA sequence. Types of epigenetic changes include DNA methylation, histone modification and non‐coding RNA sequences. Aberrant DNA methylation patterns occur in cancer cells and have been shown to participate in *SCC*.^1^ Methylenetetrahydrofolate Reductase (MTHFR) enzyme is the enzyme that catalyses the conversion of 5,10‐methylenetetrahydrofolate to 5‐methyltetrahydrofolate, a co‐substrate for homocysteine re‐methylation to methionine. It is the rate limiting step in the methyl cycle and alterations in the enzyme results in hyper or hypomethylation, both having a significant effect on folate metabolism and DNA methylation. A previous study demonstrated an association between a single nucleotide polymorphism (SNP), a genetic variation of a single base pair within the DNA, in the gene encoding *MTHFR* and the development of SCC including timing and multiplicity of tumours in a group of RTRs in a single centre in Ireland.^2^ Subsequently a functional link between this SNP to cSCC via pyrosequencing was demonstrated.^3^ The aim of our current candidate gene study was to examine further genetic alteration in the *MTHFR* gene and surrounding genes that may be relevant in the pathogenesis of skin cancer in a larger transplant population in the context of aberrant MTHFR function.

## 3 METHODS

This was a collaborative study between Beaumont Hospital, Dublin, Ireland, and the Helen Diller Family Comprehensive Cancer Centre (HDFCCC) at the University of California, San Francisco (UCSF), USA.

Adult kidney transplant populations were identified using the renal transplant databases in both facilities. Beaumont is the national renal transplant centre of Ireland and all RTRs are entered into a national database in the hospital maintained by dedicated personnel. HDFCCC provides specialized cancer care at five San Francisco medical centres and has an active clinical cancer research programme affiliated to UCSF. It has a large RTRs population attending their dermatology service whose details are also maintained on a dedicated database. All living adult RTRs with a functioning graft attending dermatology in Beaumont/HDFCCC were identified and considered for the study (*n* = 1017). Ethical approval was obtained from Beaumont hospital research ethics committee and the UCSF Committee on Human Research. Patients were excluded if they were (a) under 18, (b) had pre transplant KC, (c) diagnosis of post‐transplant KC was not clear, (d) inadequate demographics. Following consent, blood samples were genotyped for the 18 SNPs. SNPs were selected based on putative function and reported minor allele frequency (≥5%). Tagging SNPs were then selected using the HapMap databases with the overall aim was to examine SNPs in *MTHFR* and related genes that may be associated with KC in RTRs candidate gene study approach was based on previous positive association study in Ireland.^2^, ^3^

First occurrence of cSCC or BCC diagnosed after transplantation was established using histopathology diagnosis. Each patient was counted once. This included 546 patients from Dublin and 471 patients from UCSF. This data was updated on all existing patients to February 2009. Eleven SNPs in the *MTHFR* gene and seven in the overlap gene *MTHFR CLCN6* (see Table 1 for details) were evaluated using Sequenom Mass Spectrometry (further details see Table S1) and association explored by time to event analysis (from transplant to first keratinocyte cancer (KC)) was using Cox proportional hazards model. Any SNPs found to be associated at univariable level are then explored further in a multivariable regression, which included the covariates age, sex, Fitzpatrick skin type and sun exposure. A Bonferroni correction to the *p* values was used to allow for the multiple SNP tests. All the statistical analysis was conducted using Stata SE (version 13, College Station, Texas). The probability of a type 1 error was considered significant at the 5% level (*p* value < 0.05).

**TABLE 1:** Single nucleotide polymorphisms tested *n* = 18

*MTHFR* gene
rs1994798, rs1476413, rs1537514, rs2274976, rs17375901, rs1801131, rs12121543, rs1572151, s3737964, rs1801133, s17367504
*MTHFR CLCN6* gene
rs17421511, rs1804742, rs4659723, rs12759827, rs6658562, rs1050993, rs9651118

## 4 RESULTS

**FIGURE 1:**
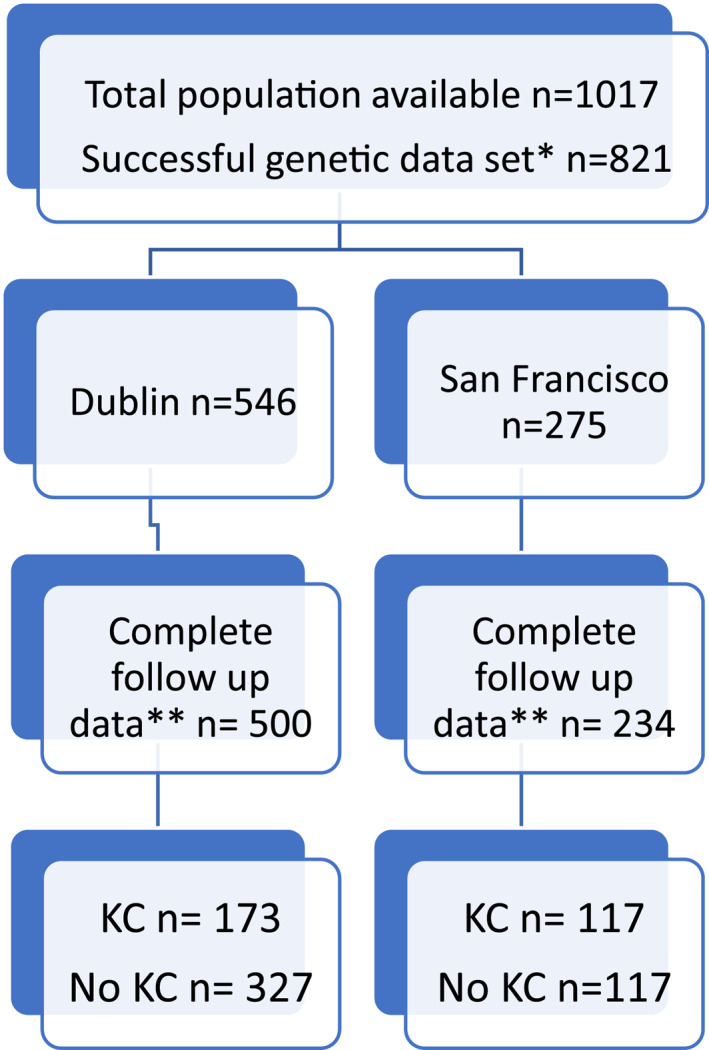
Patient recruitment numbers and distribution

Of the 1017 RTRs there was successful combined genetic data set for 821 patients. Five hundred and forty six patients from Dublin and 275 from San Francisco. Seven hundred and thirty four patients have complete follow up data and thus are included in analysis, 500 from Dublin and 234 from San Francisco (see Figure 1). The minor allele frequency of the polymorphism *MTHFR CLCN6* (rs9651118) was 10%, which was like the European Caucasian population as reported in HapMap^7^ (see Table S2a for genotyping frequencies). Overall, the group contained more males (61%), with a mean ± SD age of 48.5 ± 14.3 years and all patients were at least 3 years since transplantation. Most patients were Fitzpatrick skin type I‐III (78%; see Table 2 for demographics). The group included 290 patients with documented KC and 444 without. There were 80 with BCC and 210 with cSCC. Those with KC were more likely to be male, older at time of transplant, have Fitzpatrick skin type I‐III and have had greater sun exposure (Table 3a, 3b).

**TABLE 2:** Demographics of total transplant study cohort (*n* = 734)

	Total group	Keratinocyte cancer	No keratinocyte cancer
Number of patients	734	38% (290)	62% (444)
Sex (M/F)	61%/39% (448/286)	64%/36% (186/104)	57%/43% (191/253)
Age >50 years at time of transplant	54% (734)	69% (200)	44% (195)
Fitzpatrick skin type			
I	19% (137)	23% (62)	17% (75)
II	34% (248)	35% (101)	34% (147)
III	25% (184)	24% (71)	25% (113)
IV	16% (117)	13% (40)	17% (77)
V	5%^33^	4%^14^	4%^19^
VI	2%^14^	1%^2^	3%^12^
Time since transplant			
≥8 years	350	144 (50%)	206 (46%)
<8 years	384	146 (50%)	238 (53%)

**TABLE 3a:** Sun exposure behaviour Ireland, keratinocyte cancer V's no keratinocyte cancer *N* (%)

		Total	Keratinocyte cancer	No keratinocyte cancer
Number of patients		500	173	327
Sun exposure score	>8*	222 (44%)	114 (66%)	108 (33%)
	≤8	278 (56%)	59 (34%)	219 (67%)
**Sun exposure Score** A sun exposure score was calculated which covered time periods 0–19, 20–39, 40–59 and >60 years of patient's life. For each period, an occupational and recreational score was assigned out of maximum scores of 3 each, it was based on patient interview of hours spent outdoors during job and hobbies and the following: Whether the person lived abroad in a hot sunny climate, ever had >2 painful sunburns or used sunbeds during the time. A score of 1 was regarded as low sun exposure, 2 intermediate and 3 high sun exposure. The cumulative scores were added together so that the total sun exposure score ranged from a minimum of 2 to a maximum of 24. For further details see supplementary Table S1. The mean sun exposure score was 8 ± SD 4.4				

**TABLE 3b:** Sun Exposure behaviour San Francisco, keratinocyte cancer V's no keratinocyte cancer *N* (%)

	Total 234	Keratinocyte cancer, *n* = 117	No keratinocyte cancer, *n* = 117
Pretransplant sun exposure			
Often/Always	98 (42%)	61 (52%)	37 (32%)
Never/Seldom/Sometimes	136 (58%)	56 (48%)	80 (68%)
Pretransplant sunblock use			
Never/Seldom	105 (45%)	55 (47%)	50 (43%)
Sometimes/Often/Always	129 (55%)	62 (53%)	67 (57%)

### 4.1 *CLCN6* (rs9651118)

CC is the usual (‘non‐variant’) allele at *CLCN6.* The SNP, CT or TT, at *CLCN6* (rs9651118), was significantly associated with KC, HR 1.50, *p* < 0.00061, 95% CI 1.17–1.91, Bonferroni correction *p* value 0.001 (time to event analysis, time to first KC, Cox regression). Multivariable analysis the Irish cohort (*n* = 500) mirrored what that achieved in the univariate analysis and remained significant (HR 1.65, 95% CI 1.20–2.27, *p* = 0.002; see Table 4). Further analysis demonstrated that those with this SNP developed skin cancer years earlier than those with the CC (‘usual’) allele (Figure 1). Secondary analysis of cSCC (without BCC) of the full cohort demonstrated a HR of 1.63, CI 1.14–2.34, *p* = 0.007 for CT or TT at rs9651118. BCC was not significantly associated with this SNP although numbers were smaller (*n* = 80; HR 1.48, CI 0.92–2.40, *p* = 0.105). No significant difference was noted whether between heterozygous and homozygous that is between CT and TT.

**TABLE 4:** Risk of keratinocyte cancer with rs9651118 CT/TT in Irish RTRs *N* = 500, Cox regression multivariate analysis

Variable	Hazard ratio	[95% Confidence interval]	*p* value
rs9651118	1.649	[1.200–2.265]	0.002
Older age	1.067	[1.051–1.083]	<0.001
Male sex	1.241	[0.854–1.802]	0.257
Sun score	1.049	[1.007–1.092]	0.021
Skin type	0.771	[0.644–0.924]	0.005

### 4.2 *MTHFR* C677T (rs1801133)

A separate SNP, CT or TT at *MTHFR* C677T (rs1801133) was associated with KC in combined cohort but did not achieve statistical significance (HR 1.127, 95% CI 0.938–1.355, *p* = 0.202). Multivariable Cox regression analysis of *MTHFR* C677T (rs1801133), in the Irish cohort however demonstrated a statistically significant association (HR 1.31, 95% CI 1.05–1.63, *p* = 0.016) with KC (see Table 5). Secondary analysis cSCC (C677T) was significantly associated with SCC (HR 1.31, CI 1.02–1.68, *p* = 0.037) in Irish cohort.

**TABLE 5:** Risk of keratinocyte cancers with *MTFHR* C677T in Irish RTRs *N* = 500, multivariable analysis, Cox regression

Variable	Hazard ratio	[95% Confidence interval]	*p* value
rs1801133	1.311	[1.051–1.634]	0.016
Older age	1.068	[1.051–1.085]	<0.001
Male sex	1.283	[0.895–1.841]	0.175
Sun score	1.040	[1.000–1.081]	0.048
Skin type	0.742	[0.622–0.886]	0.001

The risk of diagnosis of KC and inheriting both SNPs (CT/TT at rs9651118 and C677T rs1801133) was significant (HR: 1.60, CI 1.16–2.23, *p* = 0.005; Table S2). The relationship between the two alleles in HapMap showed a level of linkage disequilibrium of *r* ^2^ = 0.2, implying they are inherited together 20% of the time. The genotypic variants were in Hardy Weinberg equilibrium within patient populations. No other SNPS were significantly associated with KC in our RTR group.

## 5 DISCUSSION

This study of a combined renal transplant population illustrates an increased hazard ratio of 1.5 of keratinocyte cancer, with the CT or TT variant of *Chloride transport protein 6 (CLCN6)* rs9651118. RTRs with this single nucleotide polymorphism (genetic variation at a single base pair) were found to develop cSCC years earlier than those with the usual allele (i.e., CC). No significant difference was noted between those who were homozygous or heterozygous. To the best of our knowledge, polymorphisms in this gene have not previously been reported to be associated with skin cancer although the CC variant has been shown to be protective against the development of breast cancer,^8^ lung cancer^9^ and gastro intestinal cancer^10^ in certain ethnic groups.

*CLCN6* rs9651118 is located is on chromosome one and overlaps with Methylenetetrahydrofolate Reductase cDNA, 440 000 base pairs away**.** While *CLCN6* is an intron and its exact function is unknown, MTHFR enzyme is a critical rate limiting enzyme in folate metabolism that catalyses the conversion of 5,10‐methylenetetrahydrofolate to 5‐methyltetrahydrofolate.^11^ The substrate 5,10‐methylenetetrahydrofolate is the intracellular folate required for DNA synthesis and repair, whereas the product 5‐methyltetrahydrofolate is the plasma form of folate providing the methyl group for de novo methionine synthesis and DNA methylation. Folate deficiency has been shown to impair nucleotide excision repair capacity, which is the primary repair mechanism to remove UV‐induced DNA photoproducts and alterations in MTHFR activity result in hyper or hypomethylation of DNA.^11^ Whereas excision repair affects DNA stability and hence carcinogenesis, methylation is an epigenetic example of how behaviours and environment can cause changes in gene expression that is how the body reads a DNA sequence. Aberrant methylation has been shown to be relevant in the pathogenesis of SCC in multiple ways.^1^ DNA methylation promotes (UV) radiation‐induced DNA damage, tumour suppressor genes have been shown to be hypermethylated in cSCC and global hypomethylation is thought to increase expression of proto‐oncogenes (e.g. HPV expression).^1^, ^12^, ^13^ A previous study by our group demonstrated an association between the presence of the polymorphism *MTHFR* C677T and the development of cSCC including timing and multiplicity of tumours in a group of RTRs in a single centre in Ireland.^2^ Subsequently we demonstrated a functional link between this SNP (C677T) to cSCC via pyrosequencing.^3^ These studies formed the basis of our present candidate gene approach which saw us focus on SNPs in the *MTHFR* and surrounding gene.

*CLCN6* is a chloride ion channel gene proposed to participate in chloride transport, regulation of cell volume and signal transduction. A functional study indicated that the rs9651118 CC genotype of *MTHFR CLCN6*, compared with the TT genotype, is associated with reduced homocysteine levels^14^ in a dose dependant manner. Homocysteine levels are inversely related to folate levels and consequently affect methylation. A SNP at rs9651118 may therefore exert downstream epigenetic effect on methylation via folate and homocysteine availability. Also animal studies indicate that chloride inhibits folate binding^15^ and it is possible that CLCN6 regulates chloride to facilitate folate binding, thus having an effect on folate availability. Although the frequency of the CT/TT (variant or ‘other’) allele of *MTHFR CLCN6* gene at rs9651118 was high in our study compared to the CC (‘usual’) allele, there remained a significant association with KC development in a combined American and Irish cohort which may be of benefit in future studies on skin cancer in transplant recipients. Following adjustment for multiple variables (age, sex, Fitzpatrick skin type, sun exposure) in the Irish cohort this remained significant. There was a higher hazard ratio when cSCC was analysed separately (HR 1.63) without BCC.

The burden of cSCC is increasing world‐wide and while most tumours are associated with a low risk of recurrence, nodal metastases and disease specific mortality,^16^ patients with high risk features can have a risk of local recurrence, regional and distant metastases as high as 47%.^17^ Solid organ transplant recipients have an increased risk of cSCC of approximately 60–250^4^ and an associated poorer prognosis than an immunocompetent host.^5^ Morbidly and mortality for uncontrolled locoregional disease and metastatic disease is high^18^ and indeed cSCC is a cause of death for RTRs. Thus, a need exists to identify those patients at higher risk in order to intervene earlier with possible increased surveillance, targeted preventative measures and consideration of chemoprophylaxis. Recent developments in screening approaches in transplant recipients include development of a clinical prediction score^19^ and Delphi consensus approach to screening in the US which relies on patient characteristics (sex, skin type, age at transplant, pre transplant skin cancer and recipient organ type).^19^, ^20^ Those risk factors for cSCC were strongly represented in our patients with KC as they were more likely to be male, age >50 years at time of transplant and have fair Fitzpatrick skin type. Identification of higher risk individuals via addition of a polygenic risk scores may supplement this traditional clinical risk factor approach.

Genetic studies to date have identified few syndromes associated with increased risk of SCC (including xeroderma pigmentosa, epidermolysis bullosa, Fanconi anaemia, oculocutaneous albinism and aging syndromes such as Werner syndrome), and BCC (Basal cell nevus syndrome), some higher risk genes which are primarily pigment related (MC1R, OCA2, HER2, TYR, ASIP, SCL45A2, IRF4) and HLA (*HLADQA1*).^21^, ^22^ More recently an ancestry gene effect in Hispanic and non‐Hispanic whites^23^ was demonstrated. Like CLCN6, most of these variants show small effect sizes with typical odds ratios ranging from 1.15 to 1.5. Although the total number of variants associated with KC risk is still small, there may be future benefit of using combined polygenic risk scores to identify individuals at elevated risk.^24^, ^25^

Recent therapeutic advances in KC including targeted therapy for BCCs and immunotherapy and checkpoint inhibition therapy for cSCCs hold some promise in the treatment of advanced and unresectable KCs. Immunotherapy remains challenging for solid organ transplant recipients as they risk loss of graft function and predicting patients who will respond to such treatments is a current topic of interest.^26^ Traditional therapies used to treat the spectrum of actinic field damage (topical 5 fluorouracil, 5‐FU) to metastatic unresectable cSCC (infusion of cisplatin, 5‐FU, bleomycins) exert their effects via modification of methylation and still remain in use.^27^ Indeed nicotinamide, a B vitamin, used for chemoprophylaxis of actinic keratosis, exerts gene specific effects on methylation of CpG sites in mouse models.^28^ Further studies to examine whether patients with the variant allele of *CLCN6* (i.e. CT, TT) respond better to such treatments may be of benefit. A study in patients with in colorectal cancer showed that 5‐FU based systemic chemotherapy regimens had better efficacy for methylated than unmethylated tumour suppressor genes^27^ and there is now a suggestion that intralesional 5‐FU could be effective in lower risk cutaneous SCC.^26^

We replicated findings of our earlier studies demonstrating an association between *MTHFR* C677T (rs1801133) and SCC in the Irish cohort but did not demonstrate significant association in the larger group. *MTHFR* C677T is a functional polymorphism that if present reduces the enzymatic activity.^29^ Presence of this SNP has been shown to associated with reduced serum folate in Irish and French adults.^30^, ^31^ While a recent metanalysis of C677T shows no overall association with skin cancer^32^ studies in populations with low folate status demonstrated increase risk of cSCC with this SNP which was not present if folate levels were normal.^20^ This suggests that having the C677T polymorphism alone may not be sufficient to confer increased risk but that it may need to be combined with poor folate availability or other factors, such as immunosuppression or UV exposure to increase the risk. This may explain why C677T was significant in Ireland where supplementation of food with FA is not routine and failed to demonstrate significance in the US RTR group. The US has supplemented food with FA since 1998. The role of folate in cancer is controversial.^33^ Low folate has been associated with increased risks of several cancer types (colorectum, oropharynx, oesophagus, stomach, pancreas, lungs, breast, cervix, ovary, and breast, and neuroblastoma and leukaemia)^33^ and but not others. As epigenetic influences are thought to be modifiable,^34^ this suggests a potential chemopreventive role of folate in selected populations. Further study is warranted and currently being undertaken in our unit.

We acknowledge a number of limitations to our study. As it is a point in time study it demonstrates association but not causation. Multivariable analysis was based on available clinical data on two renal transplant databases which resulted in exclusion of several patients due to incomplete demographics. Sun exposure measures differed in the two centres limiting our ability to generalize. In this is a candidate gene approach we examine 18 SNPs and acknowledge it is not a comprehensive genome wide association study. Strengths include diverse latitudes with high numbers of patients with cSCC.

## 6 CONCLUSION

In conclusion we present a SNP in the *CLCN6 MTHFR* overlap gene associated with an increased risk of keratinocyte cancer in RTRs. Those with this SNP had an increased risk of cSCC compared to those without and developed it earlier. It raises the possibility of inclusion this SNP in a polygenic risk score to identify individual RTRs at higher risk of cSCC. Within an Irish context our study suggests further examination of serum/red cell folic acid in this population.

## CONFLICT OF INTEREST

The authors declare no conflicts of interest.

## AUTHOR CONTRIBUTIONS

**L. R. Griffin:** Formal analysis; Writing review & editing. **W. L. Ho:** Data curation; Methodology. **R. J. Akhurst:** Data curation; Formal analysis. **S. Arron:** Data curation; Formal analysis; Investigation. **J. M. E. Boggs:** Writing original draft. **P. J. Conlon:** Data curation. **P. O'Kelly:** Formal analysis; Software. **A. Toland:** Data curation. **E. Epstein:** Data curation. **A. Balmain:** Investigation; Methodology. **B. Bastian:** Formal analysis. **F. J. Moloney:** Conceptualisation; Data curation. **G. Murphy:** Conceptualisation; Visualization. **M. E. Laing:** Conceptualization; Data curation; Formal analysis; Funding acquisition; Investigation; Methodology; Project administration; Supervision; Writing original draft, Writing ‐ review & editing.

## ETHICS STATEMENT

Ethical approval was obtained from Beaumont hospital research ethics committee and the UCSF Committee on Human Research. Written informed consent was obtained from all patients.

## Supporting information

Supplementary Material

## Data Availability

The data that support the findings of this study are available on request from the corresponding author. The data are not publicly available due to privacy or ethical restrictions.
