# Supplementary material for: Genetic polymorphism in Methylenetetrahydrofolate Reductase chloride transport protein 6 (MTHFR CLCN6) gene is associated with keratinocyte skin cancer in a cohort of renal transplant recipients
Source: Skin Health Dis. 2022 Feb 2;2(2):e95. doi: 10.1002/ski2.95 (PMC9168012; doi:10.1002/ski2.95)
Supplement: Supplementary file 1 — Supplementary Material [file SKI2-2-e95-s001.docx]

**Supplementary tables:**

**S1:Sun Exposure score Ireland:**

| Age (years) | 0-19 | 20-39 | 40-59 | >60 |
| --- | --- | --- | --- | --- |
| Occupational score (Maximum of 3) |  |  |  |  |
| Recreation score  (Maximum of 3) |  |  |  |  |
| Subtotals (max of 6) |  |  |  |  |
| Total (max of 24) |  | | | |

A sun exposure score was calculated which covered time periods 0-19, 20-39, 40-59 and >60 years of patient’s life. For each period, an occupational and recreational score was assigned out of maximum scores of 3 each It was based on patient interview of hours spent outdoors during job and hobbies and the following: whether the person lived abroad in a hot sunny climate, ever had >2 painful sunburns or used sunbeds during the time. A score of 1 was regarded as low sun exposure, 2 intermediate and 3, high sun exposure. The cumulative scores were added together so that the total sun exposure score ranged from a minimum of 2 to a maximum of 24.

**Table S2a.All genotyped rs9651118**

| **rs9651118** | **Frequency** | **Percent** |
| --- | --- | --- |
| CC | 38 | 4.90 |
| CT | 231 | 27.99 |
| TT | 507 | 65.34 |
| Total | 776 | 100.00 |

**Table S2b. All genotyped rs1801133**

| **rs1801133** | **Frequency** | **Percent** |
| --- | --- | --- |
| CC | 333 | 43.30 |
| TC | 334 | 43.43 |
| TT | 102 | 13.26 |
| Total | 769 | 100.00 |

**Table S3: Risk of skin cancer if have both rs9651118**CT or TT **and rs1801133**C677T, cox regression

| **Variable** | **Hazard Ratio [95 % Conf. Int.]** |  |  |  | **P value** |
| --- | --- | --- | --- | --- | --- |
| **rs9651118rs1801133**  **Older Age**  **Male Sex**  **Sun Score**  **Sun Type** | 1.604      [1.156 - 2.227]  1.068     [1.051 - 1.084]  1.262      [0.873 - 1.824]  1.040      [1.000 - 1.082]  0.747      [0.624 - 0.896] |  |  |  | 0.005  <0.001    0.217    0.052    0.002 |

**Data S1, Methods**

**SNP selection and genotyping information:**

This was a candidate gene study. The candidate gene approach allows investigation of a gene that may be involved in the condition by understanding the disease pathophysiology or a gene is in the DNA regions that have been found to be linked to skin cancer. Following our initial findings on the *MTHFR gene* we further explored related polymorphisms in *MTHFR* and the overlap gene *MTHFR CLCN 6.*

SNPs were selected based on putative function, reported minor allele frequency (5%) and the availability of a validated assay at the HDFCCC Genotyping Facility. The HapMap databases were also used to select tagSNPs.

Sequenom Mass Spectrometry was used to confirm these polymorphisms in the samples. This was undertaken within the HDFCC, San Francisco. Mass Spectrometry involves PCR amplification of the region containing the SNP of interest, an optimized primer extension reaction to generate allele-specific DNA products, and chip-based mass spectrometry for separation and analysis of the DNA analytes. A single post-PCR primer extension reaction generated diagnostic products that, based on their unique mass values, permits discrimination between two alleles. The entire process was automated including assay development, PCR setup, post-PCR treatment, nanoliter transfer of diagnostic products onto silicon chips, serial reading of chip positions in the mass spectrometer, and final analytical interpretation.

Sequenom SNP Genotyping is a mid- to high-throughput method that uses multiplex PCR coupled with mass spectrometry to interrogate up to 40 SNPs per reaction.  Among the SNP platforms available at the Core this method is more cost-effective for larger studies (greater than 10 SNPs, 100 samples). It is a good platform for sub-whole genome study applications and is widely used for fine mapping and validation studies and for routine applications that employ fixed SNP panels. The increased sensitivity of mass spectrometry over PCR-only methods makes the platform particularly sensitive and quantitative for low abundant mutations.  The validation of assays requires extra time allocated before starting the project.

The Sequenom SNP method (called iPlex Gold Genotyping) is based on multiplex PCR followed by a single base primer extension reaction.  After the PCR, the remaining nucleotides are deactivated by SAP treatment. The single base primer extension step is performed, and the primer extension products analysed using matrix-assisted laser desorption/Ionization time of flight mass spectrometry.

**source** [Labcorp | Global Life Sciences Leader in Diagnostics and Drug Development](https://www.labcorp.com/)
